# Male reproductive development: gene expression profiling of maize anther and pollen ontogeny

**DOI:** 10.1186/gb-2008-9-12-r181

**Published:** 2008-12-19

**Authors:** Jiong Ma, David S Skibbe, John Fernandes, Virginia Walbot

**Affiliations:** 1 Department of Biology, 385 Serra Mall, Stanford University, Stanford, CA 94305-5020, USA

## Abstract

**Background:**

During flowering, central anther cells switch from mitosis to meiosis, ultimately forming pollen containing haploid sperm. Four rings of surrounding somatic cells differentiate to support first meiosis and later pollen dispersal. Synchronous development of many anthers per tassel and within each anther facilitates dissection of carefully staged maize anthers for transcriptome profiling.

**Results:**

Global gene expression profiles of 7 stages representing 29 days of anther development are analyzed using a 44 K oligonucleotide array querying approximately 80% of maize protein-coding genes. Mature haploid pollen containing just two cell types expresses 10,000 transcripts. Anthers contain 5 major cell types and express >24,000 transcript types: each anther stage expresses approximately 10,000 constitutive and approximately 10,000 or more transcripts restricted to one or a few stages. The lowest complexity is present during meiosis. Large suites of stage-specific and co-expressed genes are identified through Gene Ontology and clustering analyses as functional classes for pre-meiotic, meiotic, and post-meiotic anther development. MADS box and zinc finger transcription factors with constitutive and stage-limited expression are identified.

**Conclusions:**

We propose that the extensive gene expression of anther cells and pollen represents the key test of maize genome fitness, permitting strong selection against deleterious alleles in diploid anthers and haploid pollen. Because flowering plants show a substantial bias for male-sterile compared to female-sterile mutations, we propose that this fitness test is general. Because both somatic and germinal cells are transcriptionally quiescent during meiosis, we hypothesize that successful completion of meiosis is required to trigger maturation of anther somatic cells.

## Background

Unlike multicellular animals in which germ line differentiation occurs in immature embryos, plants lack such cells destined for meiosis [1]. Growth is organized in meristems, stem cell populations that initiate organs continuously. The shoot apical meristems produce leaves and stems during vegetative growth, and a subset of these meristems switch later in development to produce flowers, a process that depletes the local stem cell population completely. Nearly all the resulting floral cells are somatic. In each maize ovary, for example, just a single cell differentiates to perform meiosis, resulting in a single embryo sac containing one haploid egg. In contrast, groups of cells in anthers differentiate for meiosis to produce large numbers of haploid pollen grains containing the sperm [1]. Although much is known about the specification of floral organs in plants, including the grasses [2], and about meiosis [3], the genes regulating the switch from mitosis to meiosis in specific cells remain largely undefined, as do the genes regulating differentiation of anther somatic cells [4].

**Figure 1:**
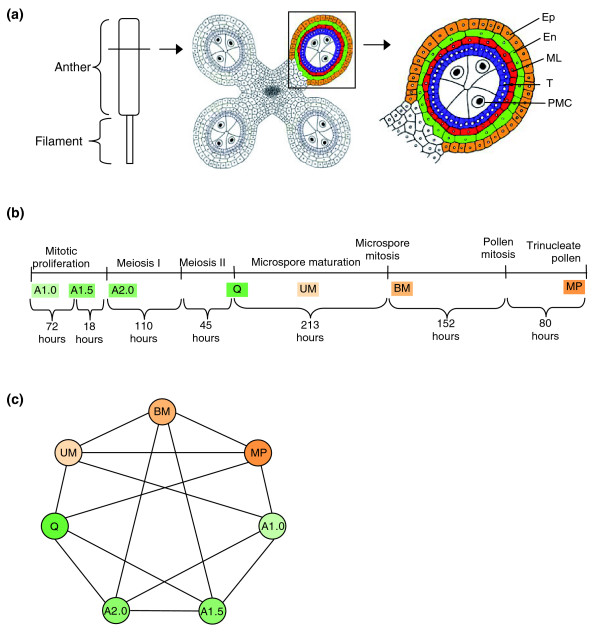
**Anther ontogeny**. **(a) **The male reproductive organ (stamen) is composed of an anther and a filament. In transverse section a mitotic (1.0 mm stage) maize anther has a characteristic four lobed structure. As cell fates are established four concentric rings of somatic cells surround presumptive meiotic cells by the 1.5 mm stage. Ep, epidermis; En, endodermis; ML, middle layer; T, tapetum; PMC, pollen mother cell. **(b) **A timeline of anther development. The top line provides developmental landmarks. Anthers were collected at the stages indicated in the second line: A1.0, mitotic anther; A1.5, anther at the cessation of mitotic proliferation with the central cells about to enter meiosis or at the beginning of prophase I; A2.0, central cells at pachytene of prophase I; Q, quartet stage of microspores, immediately post-meiotic; UM, uninucleate haploid microspore; BM, binucleate microspore; MP, mature pollen. The temporal separation between the developmental stages is indicated (in hours) below the line [6]. **(c) **Global gene expression analysis of maize anthers and pollen. Array hybridization design scheme. Four independent biological replicates with balanced dye labeling (two Cy-3 and two Cy-5) were hybridized for each stage. Each line connecting two samples represents one array hybridized with these samples. For tissue stage information see (b). The progressively darker green samples represent early anther development; the quartet stage marks the end of meiosis; the two anther maturation stages and mature pollen are in progressively darker orange.

In contrast to typical flowers containing both male (stamen) and female (carpel) reproductive organs, maize (*Zea mays *L.) has a separate ear containing carpels on a lateral branch and a terminal tassel with thousands of stamens. Within the tassel flowers (the spikelets) the carpels abort very early, hence maturing flowers contain only stamens organized in paired floral compartments (the upper and lower florets); each floret contains three stamens [2]. The stamen is a compound organ consisting of a thin filament subtending the sac-like anther; in each of the thousands of maize anthers about 500 cells initiate meiosis, ultimately producing 2,000 haploid pollen grains per anther (Figure 1a) [5]. A maize tassel can thus produce approximately 10^7 ^mature pollen grains, which are dispersed after the stamen filaments elongate to push anthers into the air through enclosing flaps of somatic floral tissues. A small opening at the anther tip permits pollen to disperse individually as from a salt shaker. Although stamens in an upper floret are developmentally ahead of the lower floret stamens by one or two days, large cohorts of stamens within upper florets along a tassel branch undergo synchronous maturation. Additionally, within each maize anther, there is near synchrony of cell differentiation [5]. The large numbers of anthers per tassel, the absence of maturing carpels, and the synchrony of anther development over a long time-frame of nearly 30 days [6] make it straightforward to collect sufficient amounts of precisely staged, upper floret anthers for biochemical analysis.

In two initial studies we used microarray hybridization to chart expression of about one-quarter of the approximately 50,000 protein coding genes of maize and found antisense transcripts for a subset of these genes. Anthers from five stages were surveyed: intact spikelets with very immature stamens (anthers <0.5 mm), 1.0 mm anthers dissected from upper florets at the rapid mitotic proliferation stage (A1.0), 1.5 mm anthers in which all cell layers are present and mitosis ceases (A1.5), 2.0 mm anthers in the leptotene-zygotene transition of meiotic prophase I (A2.0), and mature pollen [7,8]. Comparing transcriptome profiles from normal, fertile anthers to three mutants defective in cell fate acquisition or maintenance at the A1.0 and A1.5 stages yielded lists of stage-specific genes implicated as required for early steps of differentiation and likely to be characteristic of specific cell types during the first phase of anther development [8]. An unexpected observation was that transcript diversity decreased in A2.0 anthers when the central cells have just started meiosis; as more than 95% of anther cells are somatic, this result indicates that all cell types, not just the meiotic cells, are expressing fewer genes and almost no new genes compared to the previous stage. By profiling in several backgrounds it was also striking that the number of line-specific transcripts decreases progressively during anther maturation and is virtually zero in pollen [7]. Inter-species conservation of floral differentiation was evident in that many transcript types unique to anthers in maize were also expressed in rice or *Arabidopsis *flowers [8].

With the maize genome sequence now nearly complete [9], a more comprehensive microarray platform querying about 80% of the expected maize gene number was designed to more fully define the genes involved in key steps of anther and pollen ontogeny. We also wished to address the following questions: is the decrease in transcript diversity at the entry into meiosis maintained for the six day duration of this process? Are discrete transcription factors expressed during pre- and post-meiotic anther development? Given that the cell walls of several cell types are extensively remodeled during anther development, can we identify cell wall-associated processes expressed in patterns reflecting these anatomical changes?

## Results

### Design of the new 44 K maize oligonucleotide array

Transcriptome profiling of pre-meiotic anthers was previously conducted on two versions of Agilent 22 K 60-mer *in situ *synthesized arrays designed from the December 2003 maize expressed sequence tag (EST) assembly of MaizeGDB [10], containing both sense and antisense probes for selected genes. Since then, more than 500,000 long read EST sequences have become available, mainly from paired end reads of full-length cDNAs [11], increasing confidence in gene designations from contig assemblies. For this study an updated set of 60-mer probes was designed for the Agilent 44 K array format. It included validated probes from the first two maize arrays [7,8] and from anther-expressed genes detected using a spotted 70-mer array format containing probes to about 35 K maize genes [7,12]. Additional gene probes were based on release 16.0 of the TIGR Maize Gene Index [13] and cDNA or EST sequences from GenBank not yet in this assembly. The 60-mer probe sets were designed using Picky 2.0 [14]. There are 42,034 gene features representing approximately 39,000 unique sense transcript types, or about 80% of the expected gene number of maize [9], including a subset of genes with multiple probes. Approximately 500 antisense probes are also present; each gave above background signals with anther samples on the previous two versions of Agilent maize arrays [7,9]. The new array platform contains internal quantitative 'spike in' controls (see Materials and methods) that improve the accuracy of interpreting hybridization results and permit calculation of mRNA abundance.

### Transcriptome diversity during anther development and in mature pollen

As shown in Figure 1a, a maize anther consists primarily of four lobes, each with five cell types, and a small central domain containing vascular tissue and parenchyma cells. Lobes initiate with just two layers: the epidermis and an internal cell. From the onset, the epidermal cells divide anticlinally to maintain a single cell layer whereas mitotic proliferation of the internal cell occurs both anticlinally and periclinally to establish a large population. At the A1.0 stage, the discrete rings of cells characteristic of the mature anther (Figure 1a, right panel) are not yet present; however, by the A1.5 stage three days later (Figure 1b), the cell types are established and mitosis ceases. After the centrally located sporogenous cells commit to meiosis, each microsporocyte then undergoes the two divisions of meiosis to produce the quartet of resulting microspores (Q stage) over the course of about 7 days. During meiosis the anther grows slightly from 2 to 2.5 mm. Growth is accompanied by major remodeling of the original cell wall of each microspore to separate the four meiotic products, by the gradual thinning of the tapetal cell wall facing the developing microspores, and the elongation and thinning of the epidermal, endothecial, and middle layer cell walls to accommodate the increased girth of the anther in the absence of cell division. After meiosis, the uninucleate microspore (UM) stage is 9 days long; gene expression from the haploid genome could initiate during this stage and the anther grows to 4 mm through continued expansion of the pre-existing somatic anther cells. At the 5 mm anther stage, a mitotic division produces the binucleate spore (BM) containing a vegetative and a generative cell, followed six days later by mitotic division of the generative cell to produce the two sperm found in mature maize pollen (MP).

Anthers at the six developmental stages were dissected by hand, using their length as a guide. Cytological staining was performed to confirm meiotic staging for the A2.0 and quartet stages to ensure accurate pooling of samples, because there is so little anther elongation during meiosis. Pure mature pollen was collected from exerted anthers shedding pollen. The array hybridization strategies (Figure 1c) were designed as proposed by Kerr and Churchill [15]. Four independent biological replicates were used for each stage, with balanced dye labeling, on a total of 14 arrays. Such a design has been shown to minimize systematic variances associated with microarrays [15].

**Figure 2:**
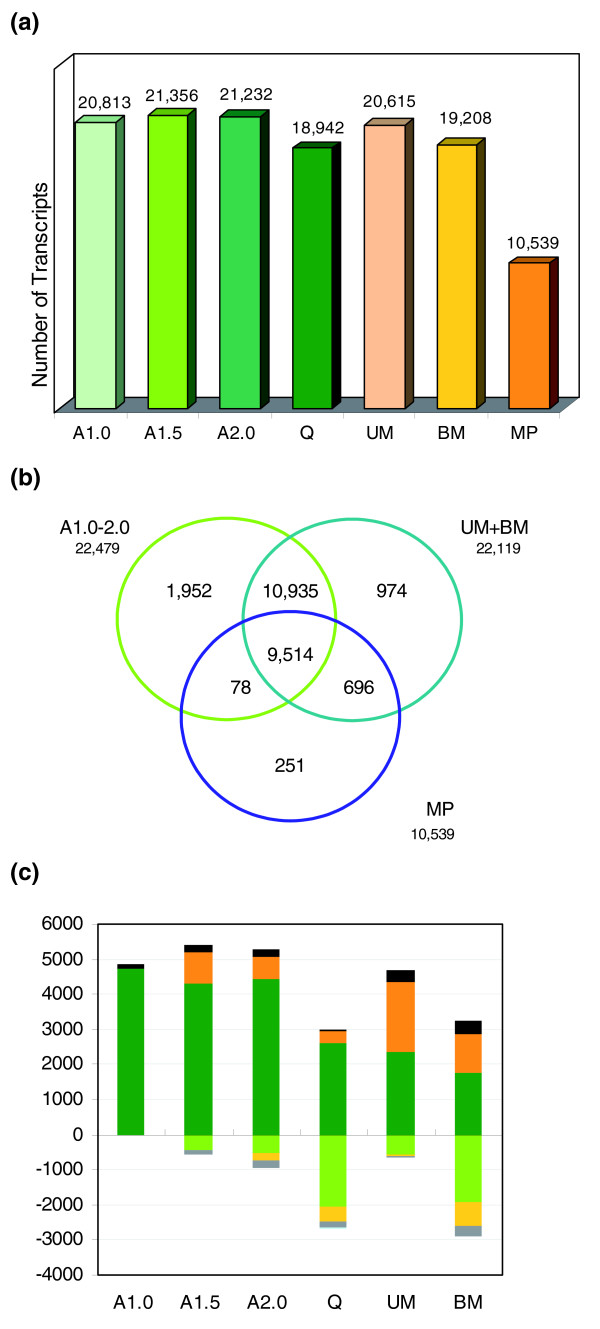
**Transcriptome constitution during development**. **(a) **Transcriptome size of the seven tissue samples. **(b) **Venn diagram showing the overlaps between anther stages (combined according to similarities in development) and pollen. The number below each stage designation is the total transcripts detected in that stage(s). **(c) **Analysis of the progression of transcriptome changes during anther development. The approximately 15,950 transcripts shared by all 6 stages are not shown. Numbers above the x-axis represent transcripts present in the indicated stage that are: stage specific (black); not present in the prior stage but shared with another stage (orange); or shared with the prior stage but missing in at least one other stage (green). Numbers below the x-axis represent transcripts present in the prior stage (from the category with a darker shade of the same color) that are not detected in the current stage. For tissue stage information see Figure 1a. Table S1 in Additional data file 1 reports the number of transcripts in each component of the histogram.

Altogether, more than 24,400 sense transcripts were found to be expressed in at least one of the six anther developmental stages plus mature pollen. As this is about 60% of the array elements (corresponding to half of the mRNA-encoding genes of maize), it is clear that male reproductive development is a highly complex process. The three early stages A1.0 through A2.0 (entry into meiosis) each express more than 20,000 transcript types (Figure 2a), followed by a dip of about 10% in transcript diversity by the end of meiosis (stage Q). Post-meiotic anthers again express about 20,000 transcripts at each stage, and mature pollen expresses about half that number.

Despite the distinctive features of early growth (stages A1.0-A2.0) compared to the UM and BM anther maturation stages that start one week later, there are only approximately 2,000 early and approximately 1,000 late group-specific transcripts (Figure 2b). These discrete phases of anther ontogeny share more than 20,000 transcript types, most of which are constitutively expressed in all anther stages, including Q, the end of meiosis. The pollen transcriptome is missing more than 10,000 transcript types expressed in early and maturing anthers. Because pollen represents the gametophytic generation in the alternation between haploid and diploid phases of the maize life cycle, we predicted that pollen might express a distinctive suite of genes, such as different members of multigene families for core cellular functions. Strikingly, mature pollen shares more than 90% of its 10,539 transcripts with all the preceding anther stages. This common set of more than 9,500 transcripts represents primarily housekeeping genes, and this evidence indicates that the same genes perform these functions in the somatic, reproductive, and haploid tissues. Only 251 genes (2.4%) of the pollen transcriptome are exclusive to that stage. Additional gametophyte-specific genes have likely already been transcribed during pollen maturation in the post-meiotic UM and BM stages; 696 transcripts (6.6% of the pollen transcriptome size) are shared between the pollen and the UM+BM stages but are not expressed earlier in anther development, and a subset of these are likely to be haploid cell-specific. Summing the pollen-specific and these shared transcripts still yields fewer than 1,000 possible pollen-specific transcripts.

The anther transcriptomes were also analyzed as a time progression (Figure 2c) focusing on the transcripts not expressed at every stage, that is, the 15,950 transcripts shared across all anther stages are not included. The transcript content of the first (A1.0) stage was set as the reference point. At this stage of rapid mitotic proliferation, there are approximately 120 stage-specific transcripts (black bar on the histogram) and thousands of other transcripts are expressed during at least one other stage (dark green bar, typically the next stage, A1.5; see Table S1 in Additional data file 1 for the gene list for each category). For both the A1.5 (cessation of cell division) and the A2.0 (start of meiosis) stages there are approximately 200 stage-specific transcripts, the loss of hundreds of transcripts present at the preceding stage (lighter shaded boxes below the x-axis), and expression of approximately 700-900 transcripts shared with subsequent stages (dark orange bars). This analysis supports the anatomical observation and previous transcriptome report that these three stages of early anther development are distinctive [5]. Furthermore, it is clear that the reduction in transcript diversity at the end of meiosis (Q stage) observed in Figure 2a reflects primarily the loss of approximately 2,700 transcripts present at the entry into meiosis (stage A2.0) with few new transcript types present. Only 34 stage-specific (black bar) and approximately 300 new types of transcripts are shared with subsequent stages (dark orange bar) at the Q stage.

One possibility to consider is that transcripts missing in a stage were slightly above the cutoff to be called present in the previous stage and are now scored as absent due to a small variance in the intensities. To examine this idea, the range of abundances of the transcripts scored as not present compared to the preceding stage was plotted (Additional data file 2). In three of the five stage comparisons, approximately 75% of the transcripts are at or above the median for the relative expression value. It is clear from this analysis that transcripts of all abundance classes are down-regulated as anthers progress from one stage to the next. Thus, the absence of specific transcript types is as valid a stage marker as the appearance of new transcript types during anther development.

In many organisms transcription is repressed in meiotic cells. The anther samples, however, consist mainly of somatic cells with a minority (<5%) of meiotic cells. Therefore, during the 7 days from entry into meiosis to the quartet stage, not only meiotic cells but also somatic anther cells exhibit a low level of activation of new gene transcription. In contrast, at the onset of anther maturation, represented by the uninucleate (UM) pollen stage, there is *de novo *expression of approximately 300 stage-specific genes and expression of approximately 2,000 genes shared with other stages, except the preceding quartet stage. Interestingly, about 600 of the carryover transcripts found in both the Q and UM stages disappear at the subsequent binucleate (BM) stage, which represents the final phase of anther and pollen maturation. At the BM stage, most of the anther volume is occupied by maturing pollen, and the transcriptome of the entire anther shows a reduction of approximately 1,400 transcripts compared to the previous stage. Collectively, these dynamic patterns of gene expression reinforce the conclusion that male reproductive development is complex in maize. The low level of new gene transcription for the one week of meiosis in the central cells indicates that the anther is an integrated system, in which activation of the anther maturation program in the somatic cell layers is contingent on the successful completion of meiosis by the central cells.

### Cluster analysis to identify co-regulated genes and Gene Ontology annotation to categorize gene types

**Figure 3:**
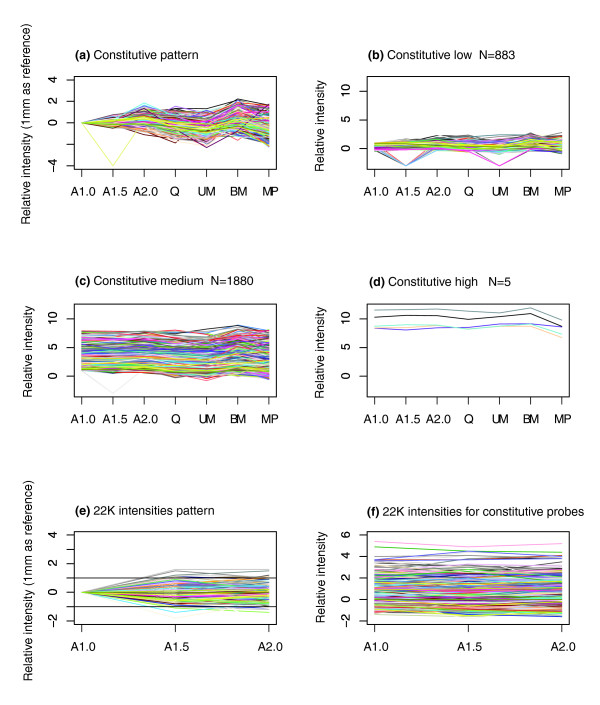
**Constitutively expressed transcripts based on k-means clustering**. **(a) **Relative intensities adjusted by subtracting the value for the A1.0 stage. Constitutive transcripts grouped by the relative intensity values at the A1.0 stage: **(b) **below 1; **(c) **between 1 and 8; **(d) **over 8. Relative expression from a previous anther study on a 22 K Agilent array for probes expressed constitutively in this study is shown **(e) **relative to the A1.0 stage and **(f) **not adjusted for the A1.0 stage.

Using the Hartigan and Wong [16] algorithm, which relies on least means squared evaluation, k-means clustering with k = 60 was performed on the gene expression profile over the entire span of anther development to identify cohorts with similar expression patterns. Missing or non-detected values were replaced with -3.0 (almost all relative expression values are larger than -2.0). Different cluster sizes were tried and highly similar results were obtained. First, the constitutively expressed genes were analyzed, as shown in Figure 3. In Figure 3a, the 2,718 genes with similar qualitative expression at all anther stages are diagrammed; because transcriptome complexity decreases by half in pollen, this stage was omitted for clustering but is displayed to provide a complete picture. Next, this cluster was subdivided into quantitative classes, as shown in Figure 3b (expression less than twice the median) through Figure 3d (expression more than 256-fold above the median). Exemplar genes drawn from these classes will serve as useful controls for quantitative PCR experiments with maize anthers [17] and for detecting deviation from normal development in male-sterile mutants of maize (see Table S2 in Additional data file 1).

As a validation test of the constitutive expression clustering, results for a set of 724 probes from a prior analysis of maize 1.0 mm, 1.5 mm, and 2.0 mm anthers on a custom Agilent 22 K microarray with sense probes to about 13,000 unique genes [8] are charted in Figure 3e. The 724 probes represent the blast hits (e-value ≤ 1e-10) of the probe sequences from the prior study against the EST sequences used for probe design of the constitutive genes determined in the current study. Data in Figure 3e are relative to the 1.0 mm stage (as was done in Figure 3a) while Figure 3f shows the actual relative expression values. Although these anthers were from a different maize background, the probes show the expected constitutive expression pattern over all three anther stages examined in the previous study.

**Figure 4:**
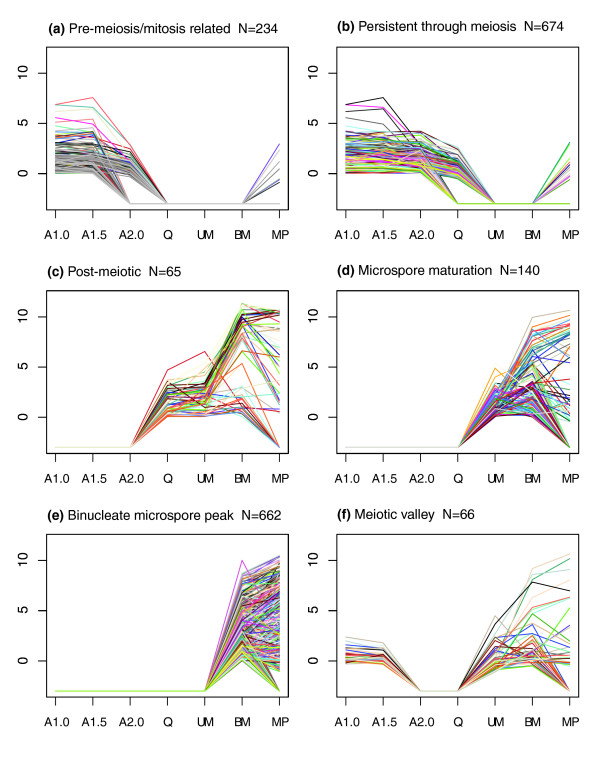
**Transcripts switched on or off between meiotic and post-meiotic stages**. **(a) **Mitosis-related: transcripts on in the A1.0 and A1.5 stages but decreasing or off in the A2.0 stage and off in the remaining stages. **(b) **Persistent through meiosis: transcripts on from the A1.0 to A2.0 stages then decreasing or off in the quartet stage and off in the remaining stages. **(c) **Post-meiotic: transcripts off from the A1.0 to A2.0 stages then on for the next three stages. **(d) **Microspore maturation: transcripts off from the A1.0 to the quartet stage then on for the two microspore stages. **(e) **Binucleate microspore peak: transcripts only on in the BM stage (ignoring pollen). **(f) **Meiotic valley: transcripts on in all stages except the A2.0 anther and quartet stages.

To address our questions about the regulation of the early and later stages of anther development, we examined the full set of clusters for specific pattern types. The Venn analysis (Figure 2b) indicates that nearly 2,000 genes are expressed only in the first three stages through entry into meiosis and about 1,000 at the UM and BM post-meiotic maturation stages. To provide more detail, genes expressed only in the first two (Figure 4a) or all three early stages (Figure 4b) and genes strictly expressed post-meiotically starting at the quartet stage (Figure 4c) or during microspore maturation only (Figure 4d) were analyzed. Two additional patterns, peak expression at the BM stage (Figure 4e) and an expression valley at meiosis (Figure 4f) were also studied.

For the pre-meiosis/meiosis-related (Figure 4a) and persistent through meiosis (Figure 4b) groups, approximately 40% of the Gene Ontology (GO) terms were associated with nucleic acid binding, protein binding and ion binding (Figure 4a) or nucleotide binding (Figure 4b and Table 1). These two groups contained several probes with similarity to genes with known roles in early anther development, including EXS, Ku70, and male meiotic chromosome organizing protein (MMD1). In the post-meiotic group (Figure 4c), the most abundant GO terms shift to the hydrolase activity, ion binding and oxidoreductase activity categories. This broad expression period contains genes with roles in cell wall degradation (for example, beta-glucanase) and one member of the phenylpropanoid pathway, flavonoid 3',5'-hydroxylase. The microspore maturation group (Figure 4d) contains 92 GO categories, with hydrolase activity, ion binding and oxidoreductase activity comprising 40.2% of the GO terms. This group contains more genes with similarity to genes involved in phenylpropanoid metabolism, including flavanone 3-beta-hydroxylase, flavonoid 3-hydroxylase, cinnamyl alcohol dehydrogenase and several different cytochrome P450 proteins. Probes in Figure 4e are predicted to contain pollen-specific transcripts as well as transcripts important for the final maturation of the anther for pollen dispersal. Surprisingly, the meiotic valley pattern (Figure 4f, transcripts present only in the A1.0, A1.5 and BM stages) contains only 66 probes. Therefore, the drop in transcript diversity during meiosis (about 2,700 transcript types) represents a major 'switch point' in that only 66 of these transcripts are re-expressed after meiosis is completed one week later. Although somatic cell division is restricted to the A1.0 and A1.5 developmental stages and the two pollen mitoses (first of the initial cell and then of the generative cell to generate the two sperm) that occur at the approximately 4 mm stage of anther elongation, we considered that perdurance of some transcripts might occur into the start of meiosis stage (A2.0). The query representing this expanded pattern (present only in the A1.0, A1.5, A2.0, and BM stages) identified 184 genes.

**Table 1:** GO term frequencies (excluding 'Unknown') for specific groups of transcripts

	Transcripts shown in Figure 4						
		
GO Term	4a	4b	4c	4d	4e	4f	Constitutive
Amine binding		0.8%			0.3%		0.2%
Carbohydrate binding		0.4%			0.7%		1.3%
Chromatin binding	1.0%	0.8%			0.7%		0.2%
Cofactor binding	1.9%	1.2%		2.2%	0.3%		3.5%
Copper chaperone activity							0.2%
Drug binding					0.3%		
Drug transporter activity	1.0%	0.8%			0.3%		0.2%
Enzyme activator activity		0.4%	2.3%		0.3%		0.2%
Enzyme inhibitor activity			2.3%	3.3%	2.1%		0.9%
Extracellular matrix structural constituent		0.4%		1.1%	0.3%		0.6%
GTPase regulator activity		0.4%	2.3%		0.7%		0.2%
Helicase activity	2.9%	0.4%					
Hydrolase activity	6.8%	8.3%	**23.3%**	**14.1%**	**11.3%**	6.3%	8.2%
Ion binding	**12.6%**	9.1%	**25.6%**	**15.2%**	**13.0%**	6.3%	**12.3%**
Isomerase activity	1.9%	0.8%	2.3%	1.1%	0.3%		1.5%
Kinase regulator activity					0.3%		0.2%
Kinetochore binding					0.3%		
Ligase activity	1.0%	2.4%		1.1%	0.3%		1.7%
Lipid binding	1.0%	1.6%		2.2%	2.1%		1.5%
Lyase activity	1.9%	0.8%	2.3%	3.3%	2.4%		1.1%
Metal cluster binding							0.6%
Microtubule motor activity	1.0%	0.8%			0.3%		
Nucleic acid binding	**10.7%**	**12.3%**	2.3%	6.5%	5.8%	**12.5%**	8.2%
Nucleotide binding	8.7%	**10.7%**	4.7%	7.6%	10.6%	**12.5%**	**11.4%**
Oxidoreductase activity	6.8%	4.7%	7.0%	**10.9%**	4.1%	6.3%	7.6%
Pattern binding		0.4%					0.2%
Peptide binding	1.0%	0.4%	2.3%				0.6%
Peroxidase activity		0.4%			1.0%		0.6%
Phosphatase regulator activity				1.1%	0.3%		0.2%
Protein binding	**16.5%**	**17.4%**	**9.3%**	5.4%	8.6%	**18.8%**	**12.7%**
RNA polymerase II transcription factor activity							0.2%
Signal transducer activity	3.9%	2.0%	2.3%	1.1%	2.4%		2.8%
Small protein conjugating enzyme activity		0.4%					0.2%
Structural constituent of cell wall	1.9%	2.4%	2.3%	3.3%	1.7%		1.7%
Structural constituent of cytoskeleton							0.2%
Structural constituent of ribosome		0.4%			0.7%		0.2%
Substrate-specific transporter activity	2.9%	3.6%	2.3%	2.2%	5.8%	6.3%	2.2%
Tetrapyrrole binding	2.9%	1.2%	2.3%	3.3%	2.1%	6.3%	1.5%
Transcription activator activity					0.3%		
Transcription cofactor activity		0.4%					
Transcription factor activity		2.4%			1.0%		0.9%
Transferase activity	7.8%	6.3%	2.3%	9.8%	**14.0%**	6.3%	9.7%
Translation factor activity, nucleic acid binding		0.4%				**12.5%**	0.4%
Translation repressor activity		0.4%					
Transmembrane transporter activity	2.9%	4.0%	2.3%	3.3%	4.8%	6.3%	1.9%
Two-component response regulator activity	1.0%	0.4%					0.4%
Vitamin binding				2.2%			0.9%
Xenobiotic transporter activity		0.4%					0.2%

Entries in bold are the most highly represented.

GO term frequencies for genes represented in Figure 4a-f are listed in Table 1 (see Table S3 in Additional data file 1 for gene lists). The last column shows frequencies for the constitutive genes charted in Figure 3a.

### Zinc-finger like proteins exhibit both constitutive and stage-limited expression

**Figure 5:**
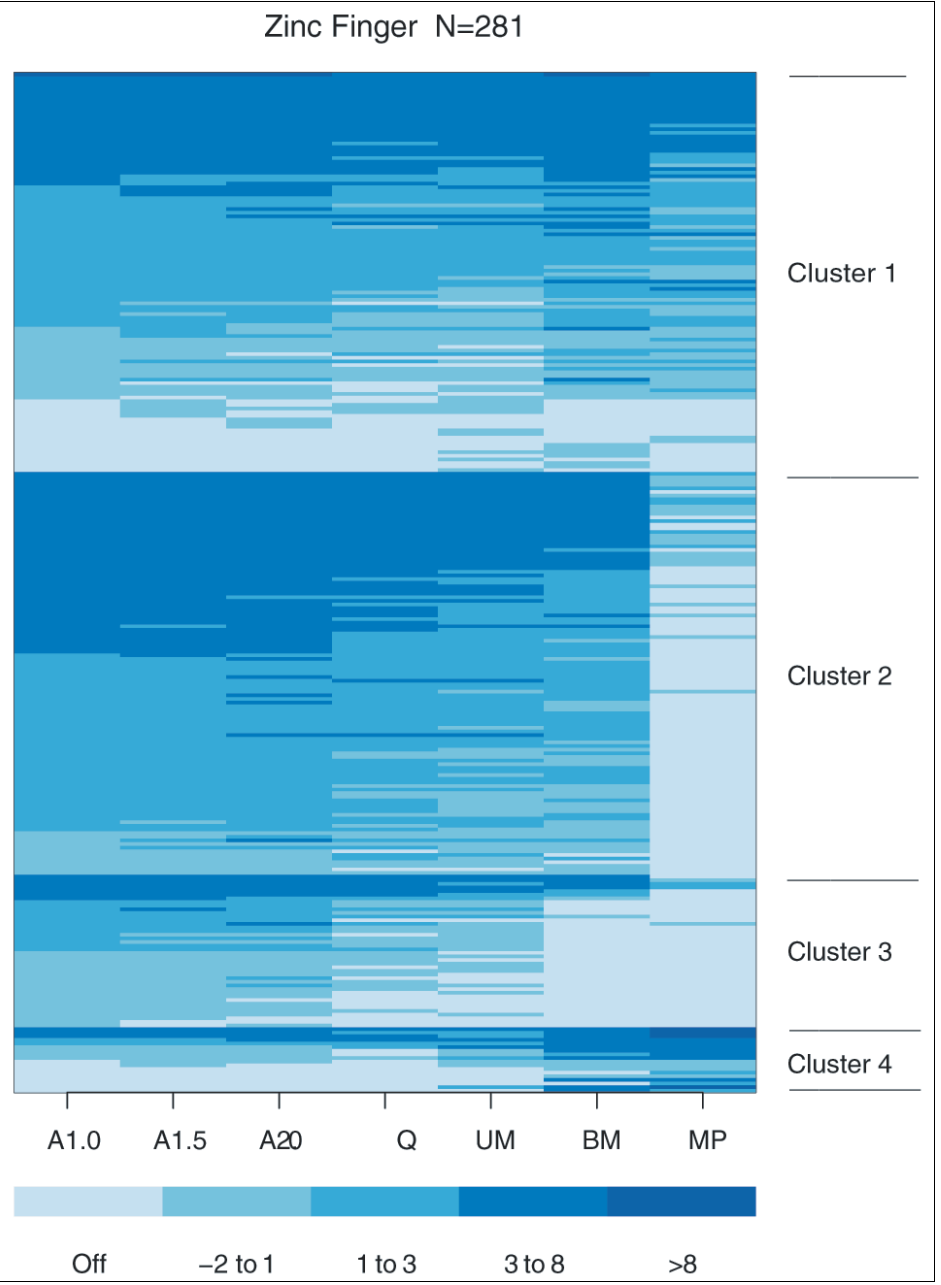
**Heatmap of relative expression values for genes similar to zinc finger proteins**. Genes are sorted by the cluster generated by k-means clustering (k = 4) then the relative expression values for each stage. Cluster 1 has mostly constitutive genes, cluster 2 is constitutive except for MP, cluster 3 has genes on in all stages except BM and MP, and cluster 4 has genes that increase in the last two stages.

Zinc finger transcription factors play important roles in developing floral tissue. In *Petunia hybrida*, seven different zinc finger proteins were sequentially expressed during anther development [18]. Several zinc finger mutants with floral tissue abnormalities have been described, including the male sterile MEZ1 meiosis-associated mutant of petunia [19] and SUPERMAN extra stamen mutants of *Arabidopsis *[20]. In this study, a comprehensive study of zinc finger protein expression was conducted. The 44 k array platform contained 281 unique zinc finger-related probes (see Table S4 in Additional data file 1). Expression analysis of these spots via a heatmap (Figure 5) identified four distinct patterns of expression: constitutive (110 genes); expressed in all stages except mature pollen (111 genes); expressed in all stages except binucleate microspore and pollen (42 genes); and up-regulated in binucleate microspore and mature pollen (18 genes). Surprisingly, the majority of zinc-finger related genes are expressed throughout most of anther development rather than being stage-limited. When the quantitative component of expression is examined, however, it is clear that within these basic categorizations there are individual zinc finger gene types that are up-regulated 4- to 64-fold at particular stages (Figure 5, darker bars at one stage or a few stages) as well as cases of similar scale for down-regulation at particular stages (lighter bars at one or two stages).

### MADS box transcription factors are expressed throughout development

The MADS box gene family exhibits diverse regulatory roles in flowering plants, including the vegetative to reproductive phase transition and determination of floral identity and aspects of stamen development [21,22]. To date, expression of three maize MADS box genes has been studied at specific stages of floral development. Both *ZmMADS1 *and *ZmMADS3 *express maximally in immature tassels (1-2 cm), approximately one week younger than the earliest samples in this study. ZmMADS1 is also expressed in microspores and mature pollen while ZmMADS3 is detectable during the later stages of development [22]. Another gene, *ZmMADS2*, is expressed highest in pollen but is also detectable in low levels in microspore and pre-dehiscent stages [19].

We extended previous studies by assessing expression of 16 (out of 34 annotated) maize MADS box genes across 29 days of development. Forty-nine probes on the 44 K Agilent array represented the 16 maize MADS-box genes (see Table S5 in Additional data file 1). At the A1.0 stage, 43 out of 49 probes were expressed but only one of these probes (zm_40370; similar to the rice MADS32 'orphan' group protein) was expressed exclusively at the A1.0 stage. Two probes (zm_13342, similar to rice OsMADS57, and zm_40357, similar to a putative maize MADS box protein) were expressed only during meiosis. None of the probes were expressed specifically in pollen. Most MADS box genes assessed were expressed constitutively, although at varying levels, as was observed for the zinc-finger like proteins.

In this study, ZmMADS1 was expressed constitutively at medium levels. ZmMADS3 was expressed highest in early development then slightly decreased through the UM stage and precipitously dropped at the final two stages to an undetectable level in mature pollen. As expected, ZmMADS2 was expressed at low levels through the uninucleate stage and increased threefold in the binucleate microspore and mature pollen stages.

Theissen and Saedler [23] have proposed a 'quartet model' of transcription regulation in which a tetramer of MADS box proteins (two dimers of two different proteins) interact with adjacent DNA binding sites via DNA bending to control gene expression at varying times in development. Thus, it is possible that the varying quantitative expression of constitutively expressed MADS box genes during anther development generates stage-specific regulatory information.

### Congruence of maize pollen array data to previous studies

Several previous reports assessed total transcript diversity in the mature pollen of angiosperms (Table 2). Transcript diversity assessed by deep cDNA-AFLP analysis led to an estimate of 12,000 genes expressed in sorghum pollen [24]. We report 10,545 maize pollen-expressed transcripts, and after a correction based on the array containing only 80% of gene types, the transcriptome of maize pollen would be estimated as 13,000, or a nearly identical estimate to a close relative. Maize and sorghum diverged about 12 million years ago [25], and the pollen transcriptome contents are very similar (data not shown).

**Table 2:** Number of transcripts in pollen estimated by different methods

Organism	Method	Estimate	Reference
*Arabidopsis*	Array	7,235	Honys and Twell [26]
*Arabidopsis*	Array	6,587	Pina *et al*. [27]
Sorghum	cDNA-AFLP	12,000	Pring and Tang [24]
Maize	*R*_0_*t*	24,000	Mascarenhas [29]
Maize	Array	10,500	This study

Honys and Twell [26] identified 13,997 mRNAs expressed in the *Arabidopsis thaliana *male gametophyte (from the microspore stage to mature pollen), including about 1,350 male-gametophyte-specific genes. In particular, 7,235 mRNA species were expressed in *Arabidopsis *mature pollen grains. A second study of this species reported 6,527 pollen transcript types [27]. These figures represent about one-third of the 22,591 probes queried. Applying this ratio to the current estimated total of 32,041 genes [28] yields 10,261 expected pollen transcript types in *Arabidopsis*. Of the 7,235 *Arabidopsis *genes identified [24], 4,407 have homologous sequences (using a tblastx *e *value <1e-40 and identity ≥ 50%) represented on this maize array. Of these 4,407 genes, 3,444 (78%) have homologous genes expressed in mature maize pollen (Table S6 in Additional data file 1). This shows substantial conservation in gene expression programs for mature pollen despite >100 million years separation of the two species. In addition, although there is not a significant correlation between the expression levels (data not shown), ranking the expression values and comparing the gene list in the first decile (344 genes) for each species shows a 30% overlap while the first quartile shows a 40% overlap. Thus, the highly expressed genes in each species show substantial overlap.

**Figure 6:**
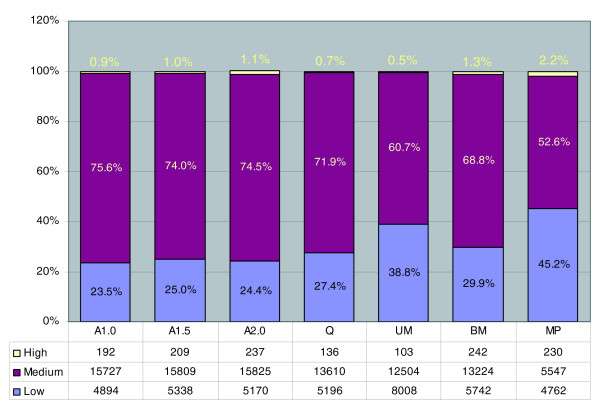
**Classification of transcript abundance classes**. Relative expression values were calculated for each stage as described in the Materials and methods based on the spike-in controls. The number of members of each abundance group is given in the included table.

In a classic study, Mascarenhas [29] used R_0_t hybridization curves to analyze maize pollen transcript diversity. His estimate of approximately 24,000 genes expressed is more than twice that based on arrays, reflecting two experimental parameters: the slow to hybridize rare transcript class can easily be over-estimated in a R_0_t hybridization because it contains short RNAs that never form stable hybrids, leading to an over-estimate of transcript diversity, while the array platform contains approximately 80% of the maize genes, with a consequent underestimation of transcript diversity. R_0_t curve structure does provide the opportunity to delineate the approximate number of transcripts present by abundance class. Maize pollen was calculated to contain approximately 240 types of high copy-number transcripts (about 32,000 copies per pollen grain), approximately 6,000 encoding medium copy-number transcripts (about 1,700 per pollen), and approximately 17,000 encoding the least abundant group (about 200 molecules per pollen) [29]. For comparison, we calculated the copy number of pollen transcripts using the expression values normalized against spike-in controls with a pre-defined copy number. If we assume, based on extrapolation from the absolute copy numbers of the spike-in controls (see Materials and methods for details), that a log-2 relative expression value of 0 corresponds to approximately 100 RNA copies per pollen grain, then 8.0 would be equivalent to about 25,600 copies, and so on. We then divided the transcriptome for each stage into three groups to parallel the R_0_t analysis. The highly abundant transcript group for pollen (relative expression value of at least 8) consists of 237 transcripts (Figure 6). The medium copy-number group has 5,547 transcripts, with relative expression values ranging from 1 to 8. These classifications are strikingly congruent with the R_0_t curve-based estimation of approximately 240 and approximately 6,000 genes for the high and medium copy-number classes, respectively [29]. We also estimated the average copy number per pollen grain for each class; that is, for the medium class there were estimated to be 1,700 copies/pollen by R_0_t curve analysis compared to 733 copies/pollen from the microarray results using the midpoint relative expression value of 2.9 (Table 3). The low copy-number group is 4,762 transcript types (Figure 6) on the arrays and about 17,000 in the R_0_t study. The average copy numbers for the low expression class is within threefold by these very different methods. We caution that some genes in the low copy-number class may not be detected on the arrays, a common caveat for microarray experiments employing tissues with multiple cell types, and some rarely expressed genes may not yet have an EST sequence and hence be missing from the array platform.

**Table 3:** Comparison of classification by transcripts abundance in maize mature pollen

		Classification by abundance			
			
	Method	High	Medium	Low	Reference
Number of transcripts	*R*_0_*t*	240	**6,000**	17,000	Mascarenhas [29]
	Array	230	**5,547**	4,762	This study
					
Average copy number	*R*_0_*t*	32,000	1,700	**200**	Mascarenhas [29]
	Array	64,702	733	**109**	This study

Estimations in strong agreement are indicated in bold.

Also of note in Figure 6, we observe that pollen contains a higher fraction (2.2%) of its transcript types in the very high abundance category, up to four times more in this class than in the six anther stages examined. The percentage of medium abundance transcripts declines after meiosis, from an average of 73% through the Q stage to an average of 65% during subsequent anther development and down to 53% in pollen; there are proportionally more low abundance transcripts in these late stages and particularly in pollen compared to earlier in anther development.

### Validation of selected genes via quantitative real-time PCR

**Figure 7:**
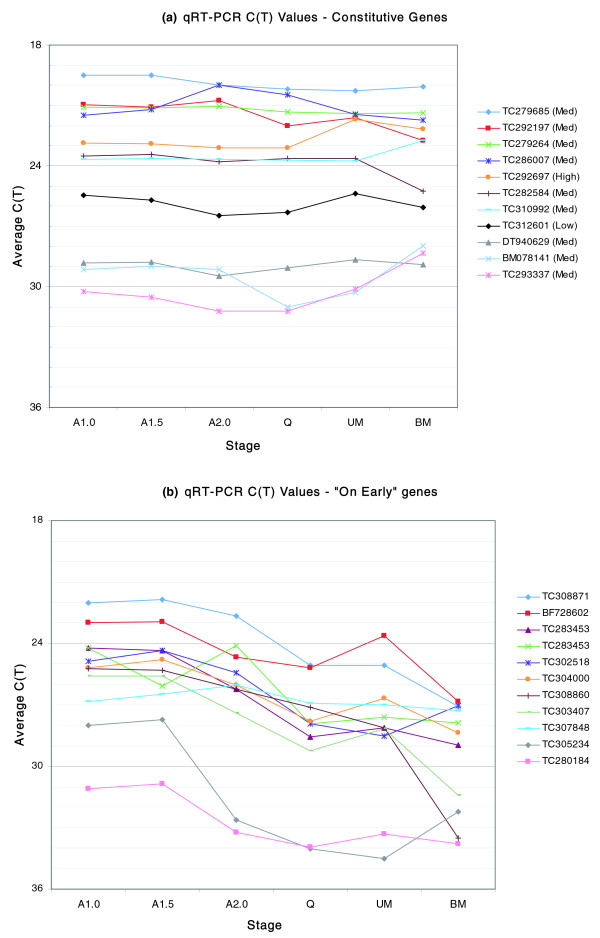
**Validation of a set of (a) 11 constitutive genes and (b) 11 'on early' genes using qRT-PCR**. Average cycle threshold (C_t_) values for three replicates are plotted for the six anther stages.

Quantitative real-time PCR (qRT-PCR) was performed to validate a subset of the constitutive (Figure 3) and 'on early' (Figure 4a) expression patterns. In total, eleven genes for each pattern (Additional data file 3) were tested at six developmental stages (A1.0, A1.5, A2.0, Q, UM and BM). The majority of the constitutive genes showed the expected expression over all six stages of development (Figure 7a). Eight of eleven genes were expressed at roughly constitutive levels at all six stages, two genes (BM078141 and TC293337) were constitutively expressed at the first three stages but then increased in expression over the final three stages, and one gene (TC288042) exhibited an eight-fold decrease in expression between the A1.5 and A2.0 stages. The constitutively expressed genes varied in expression level and spanned a 1,000-fold difference between the highest (TC279685) and lowest (DT940629) genes. In the 'on early' expression pattern, all but one of eleven genes (TC307848) exhibited a pattern consistent with that shown in Figure 4a (Figure 7b). The qRT-PCR results support both the expression patterns presented in the cluster and the cross-platform analyses, and thus provide additional evidence that the quantitative 'spike in' controls permit calculation of mRNA abundance.

## Discussion

To more fully define the genes participating in anther development, we conducted transcriptome profiling of six maize anther stages and mature pollen, capturing nearly 30 days of development, on a platform querying about 80% of the projected maize gene number. A key result is that the anther transcriptome is enormous: more than 24,000 genes are expressed by developing anthers, each anther stage expresses 19,000-21,000 genes, and even pollen with just two cell types expresses more than 10,000 genes. To date, the maize anther transcriptome is the most complex for any plant tissue. If genes not yet analyzed are expressed proportionately to the queried set, the projected maize transcriptomes are approximately 25,000 for each anther stage and 13,000 for pollen. With more sensitive detection methods, these numbers could increase further.

Because maize is a recent tetraploid, it is important to ask whether the high transcript complexity simply reflects this gene inflation. Measured mature pollen transcriptomes range from approximately 7,200/6,600 in diploid *Arabidopsis *[26,27] (by array profiling) to approximately 13,000 in sorghum, a diploid grass closely related to maize [24] (by cDNA amplified fragment length polymorphism (AFLP)). For the two species assessed by array profiling, both maize and *Arabidopsis *express about 25% of the total protein-coding genes in pollen. It will be an interesting future research question to establish the outcome of maize tetraploidization. That is, when the maize genome sequence is completed it can be determined if one progenitor is the predominant source of the current anther transcriptome or of transcripts at any stage in anther development. Also, given that anthers contain so few cell types, gene expression patterns should be further explored, whether by *in situ *hybridization or the newer method of laser microdissection of cell types, for comprehensive transcriptome analysis.

An important conclusion of the current study is that nearly all of the pollen transcriptome is shared with diploid anthers, that is, there are relatively few pollen-specific genes. Therefore, for housekeeping functions the haploid transcriptome is similar to the floral gene set. Subfunctionalization within gene families has not split the diploid floral from haploid phases of the life cycle, but it is possible that these stages express different gene family members compared to vegetative organs such as roots, leaves, and stem.

We now have a published dataset on maize leaves using a closely related 44 K platform [30]. A comparison of the normal leaf results from this new study with the 15,950 transcripts shared across all anther stages showed approximately 80% are also expressed in leaves (data not shown). The leaf data are from a platform with higher sensitivity, resulting in detection of approximately 26,000 transcript types; on the same platform, 1.0 mm anthers express approximately 32,000 genes (DS Skibbe, unpublished). Therefore, the likely anther transcriptome is even larger than documented in this study. Cross-platform validation assays for constitutively expressed genes and qRT-PCR to validate a smaller number of constitutive genes and a suite of 'on early' genes (expressed early in anther development and then not detectable by array hybridization) support the conclusions drawn from the microarray hybridization experiments. The 60-mer oligonucleotide microarray platform can quantify transcript abundance accurately over at least a 4,000-fold range (2^10 ^above the median to as low as fourfold below the median) and utilizing the spike-in control hybridization data, intensity signals can be converted to an estimate of the average transcript number per cell.

That the expression of many genes is crucial during anther and pollen development is clear from the thousands of male-sterile mutants available for maize and *Arabidopsis *[3]; importantly, most male-sterile mutants are female-fertile, particularly in maize with its separate tassel (male-only) and ear (female-only) mature flowers. Why are so many genes expressed in pollen and the supporting somatic tissues of the anther? We propose that the anther and pollen represent a critical test of genome fitness for flowering plants. Several factors underpin this test. First, both anther and carpel development are lengthy compared to leaf production during the vegetative life cycle of annual plants, and cell types perform successive roles over the course of floral development and during reproduction (Figure 1). Second, as sessile organisms, plants depend on physical forces (wind, water) or animals to deliver the microgametophyte (pollen in flowering plants) to the megagametophyte for sexual reproduction. Given the lack of specificity of these delivery mechanisms, individual species have evolved complex self-recognition systems directed to permitting pollen tube growth only within a species. Concurrently, many species have a second multi-gene system to favor pollen of a different genotype than the sporophytic flower [31]. The diversifying selection on these and other pollen specificity systems [32] could elevate the number of genes expressed in pollen, the agent for both species recognition and self-incompatibility. A third factor is that, in the anther, the majority of male-sterility traits are evident in tapetal failure [33]; this tissue supplies nutrition, structural components, and likely regulatory information to pre-meiotic cells and immature gametophytes and tapetal 'quality' is thus a key determinant of reproductive success [4].

The most important factor predates the evolution of flowers and pollen: all plants exhibit an alternation of haploid (gametophyte producing gametes) and diploid (sporophyte) generations [1]. Unlike animals in which nearly all genes are silent in the haploid gametes, the gametophytes of plants are self-sufficient in transcription and many other processes. In the least advanced forms, such as algae and mosses, the haploid phase dominates the life cycle and the sporophyte is a transitory phase. In the ferns, gymnosperms, and angiosperms (flowering plants) the sporophyte is the dominant phase; however, even in flowering plants the microgametophyte (pollen) is separated from the sporophyte nutritionally after acquisition of thick pollen coatings following meiosis. Single (haploid) copy genes required for housekeeping functions such as metabolism, macromolecular synthesis, organelle and membrane maintenance, and mitosis sustain pollen maturation prior to dispersal and even more critically fuel growth of a pollen tube (approximately 20 cm in the case of maize) to deliver sperm to the ovary. The stringent haploid sufficiency test for about 25% of the genome at each generation purges plant genomes of many deleterious alleles that cause no phenotype in the heterozygous condition but are highly deleterious or lethal when homozygous. For this test to be an advantage to the diploid organism, haploid pollen must express genes that are also expressed in the dominant phase of the lifecycle. This is the case for the approximately 95% overlap between pollen and the developing anther. The absence of a haplosufficiency test in animals results in substantial genetic load and presents the human population with the tragedy of heterozygous carriers for embryo and juvenile-lethal diseases.

A new component of the sufficiency test was uncovered in this study. The week long meiotic period in maize is accompanied by little new gene expression in the entire anther. Therefore, the complement of transcript types in both pre-meiotic central cells and the supporting somatic cells must suffice to sustain anthers for an extended period. Future experimental analysis could resolve whether the appearance of so few new transcript types during this long interval is actually more profound - is transcription repressed for all or most genes in most cell types? If true, then the mRNAs made prior to the onset of meiosis must persist throughout this key developmental period without replacement synthesis in either the meiotic or somatic cells. Because both mitosis and meiosis involve extreme condensation of the chromosomes, it is not surprising that transcription would be reduced for cells conducting these processes; however, the anther somatic cells are post-mitotic when meiosis starts. The mechanisms limiting new gene transcription in the somatic cells and then activating robust new gene transcription as the central cells exit from meiosis are completely undefined and are, therefore, a challenge for future investigation.

A second important biological insight from our analysis at the entry and exit from meiosis is that the anther functions as a single functional unit, an integrated system in that global controls are exerted on gene expression. In prior work we used developmental mutants of maize to test two models of anther cell fate specification: a lineage model in which the highly patterned cell division progression sets cell fate and the contrasting model of fate setting just prior to meiosis through cell signaling. The weight of evidence - particularly the phenotypes of the *mac1 *and *am1 *mutants - favors the late decision model over the lineage model. Signaling that meiosis has started and later finished is now implicated to achieve transcriptional stasis during meiosis, implicating coordinating mechanisms within the anther to communicate meiosis start and completion. As anther lobes lack vascular tissue, these signals are likely to move from cell to cell.

Considering that more than 20,000 genes are expressed at most anther stages, many transcription factors must be required to program this expression. Given that the vast majority of probes from the two transcription factor families investigated exhibit constitutive or broad patterns of expression across multiple stages, a third message is that a simplistic model of stage specific transcription factors is inadequate. Many more transcription factors show substantial (4- to 64-fold) quantitative changes, a small subset of which are stage-specific. Combined with the possibility of post-translational modification of protein to modulate activity state, it seems highly likely that development is orchestrated by quantitative changes in the abundances of suites of transcription factors.

During anther ontogeny, cell walls are built and remodeled during mitotic proliferation and cell expansion and maturation. Most dramatically, as the microspores mature into pollen each acquires a thick wall; the outer (exine) wall consists mainly of products secreted by the neighboring tapetal cells while the inner wall (intine) is synthesized mainly by the haploid gametophyte. Tapetal cell walls are initially thick but selective degradation during meiosis eliminates nearly the entire wall facing the microspores, presumably facilitating secretion of materials. In the other somatic cell types, walls are remodeled during the expansion from 1.5 mm to the final approximately 5 mm length, in the absence of cell division, resulting in a switch from cuboidal to rectangular cells. Within a partial catalog of 131 cell-wall associated transcripts (Table S7 in Additional data file 1), several show stage-specific expression (for example, pectin methylesterase and pectinase are highly expressed only in the BM and mature pollen stages). This catalog represents a first step in acquiring a comprehensive view of cell wall building and remodeling during anther and pollen development.

## Conclusion

Although simple in anatomy, maize anthers express an astonishingly large number of genes over the one month of development surveyed. It is projected that at least half of protein-coding genes of maize are expressed over the six anther stages analyzed and that at least 40% of the genome is expressed at any given stage. We propose that this high level of gene expression diversity is a 'genome fitness' test for the plant. During the one week of meiosis by the central lobe cells, the entire anther is relatively quiescent in terms of gene transcription activation: neither the somatic cells (95% of the anther) nor the meiotic cells are expressing new genes. Post-meiotically, however, diverse new gene types are expressed during anther and pollen maturation. We found few cases of stage-specific transcription factors, although instances of stage-specific quantitative modulation are common. It has been proposed that the haploid gametophyte could have a unique transcriptome compared to the diploid plant; in this study 95% of pollen-expressed genes are shared with the diploid anther. As a consequence, the haploid-sufficiency test encompassing more than 10,000 genes in the flowering plants examined to date can be effective in eliminating deleterious alleles expressed in pollen that result in lethality or poor growth. Our results indicate that this test assesses genes critical for diploid floral development as well.

## Materials and methods

### Biological materials and tissue collection

The W23 line carrying the *bz2 *mutation (lack of anthocyanin pigment accumulation in the vacuole) is maintained in the Walbot laboratory by self-pollination. The materials were grown at Stanford University in the summer of 2005, and tissue collection was done as described in Ma *et al. *[4]. Cytological classification was performed as described in Chang and Neuffer [5] for acetocarmine staining of meiotic stages, or Kindiger [34] for hematoxylin staining of microspores and pollen to ensure that the anthers collected were at the correct developmental stages (Figure 1a). Up to five replicate samples were obtained for each stage.

### Array design and data analysis

The custom 44 K Agilent maize array was designed based on the previous two versions of Agilent maize arrays [4,5], the University of Arizona spotted oligonucleotide maize array [4], and release 16.0 of the TIGR Maize Gene Index [13]. The set of 60-mer oligonucleotide probes was designed using Picky 2.0 [9] with the following parameters: G+C content 40-70% and minimum mismatches of 15 bases between non-targets. Handling of the arrays and data analysis were carried out as described previously [4] with the following modifications. Target cRNA was prepared and labeled with either Cy-3 or Cy-5 dye from 0.5 μg of total RNA using an Agilent Low RNA Input Fluorescent Linear Amplification Kit Plus with spike-in controls (Agilent, Santa Clara, CA, USA). Array hybridizations and washing were carried out according to Agilent Gene Expression Protocol 4.0. Significant improvements in signal to noise were achieved by hybridizing for 17 h at 65°C instead of the 60°C used in previous protocols and by performing the first wash at 37°C instead of room temperature.

Array hybridization signals were extracted and normalized with Feature Extraction 9.1 (Agilent) using standard locally weighted linear regression algorithm (lowess) with a universal error model as described previously using open-source packages [4]. Improvements in both the software and array hybridization protocols, especially with the addition of the 30×10 spike-in controls, increased our confidence in Feature Extraction. Each slide was inspected manually to flag the low number of spots that were contaminated by small debris left after washing; such spots were not included in subsequent analyses. Saturated spots or non-uniform outliers (as determined by Feature Extraction) were also removed. For a given probe to be classified as 'present', we required at least three out of four hybridization signals to be 'well above background' (99% confidence), a statistic generated by Feature Extraction based on the distribution of local background signals. Dye-normalized signals from Feature Extraction were normalized again to spike-in controls (E1A_r60_a97 for Cy-3 signals, and E1A_r60_n11 for Cy-5 signals, each with a relative copy number of 0.5; log-2-base) so that the expression values could be compared from slide to slide. Finally, outliers were detected and removed with a Grubb's test across the four biological replicates, and the averaged relative expression value used for subsequent analysis. The relative expression value was calculated as log-2(Normalized intensity/Median intensity of hybridizing probes on the array) and then averaged across the biological replicates. Based on the experiment design, results were analyzed as in a single-channel hybridization experiment and without performing a differential expression analysis between samples. Original transcriptome data generated in this study can be accessed at the Gene Expression Omnibus (GEO) under accession [GEO:GSE12579].

### K-means clustering

Clustering was performed in the R programming environment [35] using the kmeans function (parameters: iterations = 150, algorithm = Hartigan-Wong) within the cluster package [36]. The relative expression value for the A1.0 stage was subtracted from all stages prior to clustering. The assigned cluster was used for charting both the qualitative and quantitative data.

### Heatmap

The zinc finger heat map was generated in R [35] using the 'image' and 'axis' functions. The data were sorted by cluster and expression values for each stage prior to generating the heatmap.

### Gene Ontology analysis

GO terms were assigned to the microarray probes using tools at the Agbase website [37]. Probes were matched to proteins locally using blastx and the resulting protein list was submitted for GO annotation at Agbase (GORetriever tool). Probes still lacking GO annotation were submitted to Agbase for combined protein matching and GO annotation (GOanna tool).

### Estimation of RNA copy numbers from array hybridization signals

The spike-in controls from Agilent are a mixture of 10 single-stranded RNA (ssRNA) sequences of approximately 500 nucleotides in length. In particular, the spike-in controls used for the final log-2 normalization (E1A_r60_a97 for the Cy-3 labels, and E1A_r60_n11 for Cy-5 labels) were present at a concentration of 100 pg/μl in the original mix. The dilution was 1,600 fold, resulting in 0.0625 pg starting control approximately 500 nucleotides ssRNA; for this length 1 μg is equivalent to 3.75E12 molecules (Ambion Molecular Biology Tables); consequently, about 234,000 copies of control ssRNA were used in the initial amplification reaction. Thus, a log-2 relative expression value of 0 would be equivalent to 234,000 copies of an RNA species in the starting materials, which was 500 ng of total RNA extracted from mature pollen grains.

Mascarenhas [29] estimated that individual maize pollen grains contain 352-705 pg of total RNA. We assumed that one pollen grain contains 500 pg of total RNA. Therefore, the 500 ng of total RNA used in our starting materials would be from 1,000 pollen grains. To estimate the average length of maize mRNA from the TIGR maize gene index assembly (version 16), we took only the 36,625 TC sequences (tentative contigs) as they should be closer to the distribution of lengths in the real transcriptome than singlet ESTs. The mean length calculated from this collection is 1,015 nucleotides. Comparing this to the 500 nucleotides length of the spike-in controls, we used a scaling factor of 2 to arrive at an estimate of 117 copies/pollen grain (234,000 copies/2/1,000 pollen). Again, for simplicity, we used for the final estimation 100 RNA copies/pollen if a probe has a log-2 relative expression value of 0. All the copy numbers thus should be viewed as an average. Note that it is possible to calculate the 'absolute' per pollen copy number for any given transcript of known size.

### Quantitative real-time PCR

Approximately 2 μg of DNase-treated total RNA for the A1.0, A1.5, A2.0, Q, UM and BM was reverse transcribed with an oligo-dT primer using the SuperScript III first-strand synthesis system for RT-PCR as recommended by the manufacturer (Invitrogen, Carlsbad, CA, USA). Primers were designed using Primer3 and synthesized by Illumina (San Diego, CA, USA). The complete list is presented in Additional data file 3. qRT-PCR was performed on an OPTICON2 sequencing detection system (MJ Research, now a part of Bio-Rad, Hercules, CA, USA). A 50 μl reaction mixture containing 25 μl of iQ SYBR Green Supermix, 0.5 μM each primer, and approximately 10 ng of cDNA was amplified using the following cycling parameters: 95°C for 5 minutes, followed by 40 cycles of 95°C for 10 s, 60°C for 1 minute and a plate read. Upon completion of the program, a melting curve analysis was performed from 55 to 95°C with a read every 0.5°C with a 10 s hold. Each of the 22 genes was tested and confirmed to amplify a single band via melting curve analysis and agarose gel electrophoresis. The cycle threshold numbers (C_t_) at which each sample reached the threshold fluorescence level for each type of PCR product were determined for all samples using the OPTICON2 software.

## Abbreviations

A1.0: anther 1 mm stage (mitotic); A1.5: anther 1.5 mm stage (pre-meiotic); A2.0: anther 2 mm stage (meiotic); AFLP: Amplified Fragment Length Polymorphism; BM: binucleate microspore; EST: expressed sequence tag; GO: Gene Ontology; MP: mature pollen; Q: quartet stage (end of meiosis); qRT-PCR: quantitative real-time PCR; ssRNA: single-stranded RNA; UM: uninucleate microspore.

## Authors' contributions

JM prepared the samples, performed the microarray hybridization, and conducted the initial data analysis as part of his PhD thesis work. Subsequent analysis and manuscript preparation was performed by DSS, JF, and VW. DSS performed the qRT-PCR validation.

## Additional data files

The following additional data files are available with the online version of this paper. Additional data file 1 contains seven supplementary tables of genes as follows. Additional data file 2 is a box and whisker plot figure showing relative expression values of transcripts present in one stage that were 'missing' in the following stage (that is, the list of probes in Figure 2c below the x-axis). Additional data file 3 is a table listing the primers for the 22 genes used in the qRT-PCR validation of the microarray results.

## Supplementary Material

Additional data file 1 Table S1: Figure 2c detail. Table S2: Figure 3 detail. Table S3: Figure 4 detail. Table S5: MADS box genes. Table S6: genes found in both maize and *Arabidopsis thaliana *pollen. Table S7: cell wall associated genes. Click here for file

Additional data file 2 Relative expression values of transcripts present in one stage that were 'missing' in the following stage (that is, the list of probes in Figure 2c below the x-axis). Click here for file

Additional data file 3 Primers for the 22 genes used in the qRT-PCR validation of the microarray results. Click here for file
