# Supplementary material for: Male reproductive development: gene expression profiling of maize anther and pollen ontogeny
Source: Genome Biol. 2008 Dec 19;9(12):R181. doi: 10.1186/gb-2008-9-12-r181 (PMC2646285; doi:10.1186/gb-2008-9-12-r181)
Supplement: Additional data file 2 — Relative expression values of transcripts present in one stage that were 'missing' in the following stage (that is, the list of probes in Figure 2c below the x-axis). [file gb-2008-9-12-r181-S2.doc]

Supplemental Figure 1
